# Design of Mucoadhesive Strips for Buccal Fast Release of Tramadol

**DOI:** 10.3390/pharmaceutics13081187

**Published:** 2021-07-31

**Authors:** Nayla Francine Garcia Pastório, Camila Felix Vecchi, Rafaela Said dos Santos, Marcos Luciano Bruschi

**Affiliations:** Laboratory of Research and Development of Drug Delivery Systems, Postgraduate Program in Pharmaceutical Sciences, Department of Pharmacy, State University of Maringa, Maringa 87020-900, PR, Brazil; naylafrangarcia@gmail.com (N.F.G.P.); camilaf.vecchi@gmail.com (C.F.V.); rafaelasaids@gmail.com (R.S.d.S.)

**Keywords:** thin films, mucoadhesion, fast dissolving, development, mechanical properties, polymeric systems

## Abstract

Tramadol hydrochloride is a synthetic analogue of codeine and shows activity on the central nervous system as an opioid agonist and inhibitor of serotonin and norepinephrine reuptake. It has been used for controlling moderate to severe pain. Mucoadhesive fast-dissolving films can present greater drug availability and patient acceptance when compared to the systems of peroral administration. The films were prepared using the solvent casting method with ethylcellulose, polyvinylpyrrolidone and poly(vinyl alcohol). The effect of each polymer concentration was investigated using a 2³ factorial design with repetition at the central point. The formulations were subjected to physicochemical, mechanical, ex vivo mucoadhesive and in vitro drug release profile analysis. These properties were dependent on the polymeric composition (independent factors) of each system. The optimized formulations showed good macroscopic characteristics, improved resistance to bending, rigidity, rapid swelling up to 60 s, improved mechanical and mucoadhesive characteristics, and also fast dissolving and tramadol release. The optimized formulations constitute platforms and strategies to improve the therapy of tramadol with regard to availability at the site of application, considering the necessity of rapid pain relief, and show potential for in vivo evaluation.

## 1. Introduction

Pain may be defined as an unpleasant sensory and emotional experience accompanied with actual or potential tissue damage [1,2]. The single and most reliable pain indicator is the self-report of the patient. Biological, psychological, and social factors can influence this personal experience to varying degrees [2]. Individuals learn about the concept of pain through their life experiences. Thus, pain plays an adaptive role, which may have adverse effects on well-being and psychological and social function [3,4]. This uncomfortable feeling may be classified into acute or chronic [2,3,5] and emotional or sensory pain [4]. Recommendable appropriate analgesics for emotional pain are anxiolytics (minor tranquilizers), antidepressants, and antipsychotics (major tranquilizers). However, for sensory pain, the recommendations are different. Anticonvulsivants and antidepressants are recommended for neuropathic pain, while non-steroidal anti-inflammatory drugs (NSAIDs), n-acetyl-para-aminophenol, acetyl salicylic acid, steroids and opioids are indicated for nociceptive pains, such as somatic and visceral pain [4].

Tramadol is a synthetic 4-phenyl-piperidine analogue of the opioid drug codeine that acts on the central nervous system as an agonist, by inhibition of serotonin and norepinephrine reuptake, while its metabolite *o*-desmethyltramadol acts on the μ-opioid receptor [6,7,8]. The chemical formula of tramadol is 2-(dimethyl amino)-methyl)-1-(3′-methoxyphenyl) cyclohexanolhydrochloride, and it was first synthesized in 1962 by the west German pharmaceutical company Grünenthal GmbH (Stolberg, Germany), introduced to the market under the trade name Tramal^®^ in 1977. Its potency is about one-tenth that of morphine, but is preferred due to it being safer than the latter [7,8].

The discovery of tramadol revolutionized the pain medication market. This active pharmaceutical ingredient (API) is utilized for the treatment of both acute and chronic pain of moderate to severe intensity. Compared with other opioid analgesics, it does not cause respiratory depression and addiction [6,7]. Tramadol displays different characteristics from opioids available on the market, namely its dual action mechanism, which allows it to maintain a large part of its effectiveness. It exhibits agonistic activity to the μ-opioid receptor (MOR) and the central GABA catecholamine and serotonergic receptors [7]. Therefore, it is mainly utilized in the management of chronic pain and as first-line API in the treatment of joint, muscle and wound pain, including the therapy of postoperative or orthopedic injury induced acute pain [6,7,9].

Formulations containing tramadol are available for peroral administration (tablet, capsule and syrup), parenteral administration (intramuscular and intravenous), and local administration (cream, gel and ointment) [6,7,8]. It is an API of high solubility and permeability (biopharmaceutical classification-I) [6,7,10]. This characteristic enables the dissolved drug molecules of tramadol to permeate through the mucosal membrane and to reach the microvasculature. Therefore, the API is rapidly absorbed and has a half-life of approximately three hours, often requiring a larger number of doses to promote the proposed therapeutic effect [6,7,8,9,10]. For the effective management of the therapeutic condition, in the case of patients with orthopedic problems, presenting problems in the musculoskeletal system, ligaments and joints, it is very common to administer several doses of tramadol in order to maintain an adequate and constant concentration for a certain period of time [9]. For example, when administered orally, tramadol is utilized at 50–100 mg every 4–6 h with or without food. The maximum dose of this API is 400 mg/day, and it is intravenously and intramuscularly used in severe pain with a dose of 50–100 mg every 4–6 h [6,7,8].

Considering the degree of invasiveness of the parental route of administration, as well as the need for frequent doses through oral administration, patient compliance with treatment and therapy may be impaired. In addition, the fast effect/action of tramadol against pain is necessary in cases of acute pain. Therefore, it is necessary to optimize the administration route and the development of platforms for the modified and/or controlled release of tramadol in order to overcome these inconveniences.

Transmucosal route of drug delivery can offer important advantages over the other administration routes for systemic delivery, including the possible bypass of the first-pass effect and avoidance of presystemic elimination within the gastrointestinal tract [11,12]. The mouth is an accessible site for the administration of pharmaceutical systems, and the buccal drug administration is widely accepted for potent medicines for the clinical situations associated with discomfort and severe pain [9,11,12].

Mucoadhesive systems can provide intimate contact between a pharmaceutical system and the absorbing tissue, which can result in a high drug concentration in a local area and high drug flux through the absorbing tissue [11,12,13]. Different dosage forms showing mucoadhesive properties intended for buccal administration have been proposed, such as tablets, patches and adhesive semisolid systems [11,14,15]. These systems must keep intimate contact with the mucosal membrane, facilitating both mucus interaction and permeation and epithelial absorption as well [12]. Patches can be designed to constitute simple erodible and no erodible mucoadhesive films and to provide either unidirectional or multidirectional drug release [11]. These systems can be developed for prolonged or for rapid release.

Fast-dissolving films or strips have gained acceptance and popularity due to their rapid disintegration/dissolution, and as they can be self-administered even without water or chewing. They can also overcome difficulties of administration associated with solid dosage forms for geriatric and pediatric patients [15,16].

Mucoadhesive buccal systems containing tramadol have been proposed. Despite them having displayed a prolonged release, the rapid effect of the drug has shown to be impaired [9,17]. In previous studies, we have developed mucoadhesive films comprising polyvinyl alcohol (PVA), polyvinylpyrrolidone (PVP), and poloxamer 407 for pharmaceutical applications [18]. They showed good mucoadhesive properties and improved performances for fast drug delivery [19,20]. Therefore, the objective of this work was to develop a mucoadhesive polymeric platform in the form of a fast-dissolving strip composed of PVA, PVP, and ethylcellulose for buccal delivery of tramadol. These polymers are already used in the composition of many pharmaceutical dosage forms and exhibit good performances, safeness and cytocompatibility [18,19,20]. Design, technological preparation, physicochemical and mechanical characterization, in vitro drug release, and mucoadhesiveness evaluations of the system were performed.

## 2. Materials and Methods

### 2.1. Materials

Tramadol hydrochloride was purchased from Cadila Healthcare Limited (Gujarat, India) and ethylcellulose (EC; Surelease^®^ grade E−7-19040) was from Colorcon (Cotia, SP, Brazil). Polyvinylpyrrolidone (PVP, mm = 111.14 g/mol) was purchased from Labsynth (Sao Paulo, SP, Brazil) and poly(vinyl alcohol) (PVA, mm = 44.05 g/mol) was received from Neon (Sao Paulo, SP, Brazil). Absolute ethanol was received from Vetec^®^ (Duque de Caxias, RJ, Brazil) and methanol (analytical grade) was purchased from Merck (Sao Paulo, SP, Brazil). Ultra-purified water utilized in all experiments was obtained from an Evoqua^®^ apparatus (Günzburg, Germany).

### 2.2. Design and Preparation of Systems

A full factorial design 2^3^ was employed to investigate the influence of PVA (X_1_), PVP (X_2_), and EC (X_3_) concentrations that have a significant influence on the study response. The independent variables (PVP, PVA, or EC) were evaluated at two levels, low (−) or high (+), as displayed in Table 1. Moreover, two central points (F9 and F10) were also evaluated for detection of curvature and errors associated with isolated effects or the interactions among them, totaling ten formulations.

They were prepared by dispersing the polymers and drug in a suitable solvent (purified water or absolute ethanol). PVA was dissolved in purified hot water at 70 °C and PVP was dissolved in ethanol under magnetic stirring. EC and the drug were also dispersed separately in purified water. A suitable amount of tramadol hydrochloride with 10% (*w*/*w*) of drug in each formulation was dissolved in purified water, at room temperature, and under magnetic stirring until complete dissolution. This aqueous solution of tramadol hydrochloride was added to the PVA solution (at 25 °C), under stirring for 5 min. Afterwards, the EC and PVP solutions were also added under agitation for the time required until complete homogenization. The final dispersion was poured into circular plates for drying in a circulating air oven at 50 °C for 4 h. Afterwards, the films were removed from the molds and macroscopically evaluated for integrity, homogeneity, flexibility, presence of air bubbles, color and touch adhesion [18]. They were kept dry, at room temperature, and protected from light until the further analyses.

### 2.3. Analysis of Thickness and Density

The film samples were measured as thickness at five random sites using a micrometer. Three samples from each formulation, measuring 1 cm^2^, were oven-dried at 40 °C for 20 h, and weighed using analytical balance. Afterwards, the density of each film sample was determined according to the following equation [18,21,22,23]:

(1) $$D=\frac{m}{A\cdot h}$$

where *D* is the density (g/mL), *m* is the mass (g), *A* is the surface area (mm^2^), and *h* is the thickness (mm) of film sample. At least three replicates were carried out to estimate the inherent variability of each analysis.

### 2.4. Determination of the Swelling Index

The analysis of moisture uptake capacity of formulations was determined using samples of 100 mm^2^ area. The strips were dried using a fan-assisted oven, at temperature of 40 °C up to constant weight. Afterwards, each sample was weighed and immediately submerged in purified water. After 60 s, the excess water was gentle removed using an absorbent paper, and the film sample was weighed again. The following equation was utilized to determine the swelling index (SI) [22]:

(2) $$SI\;\left(\%\right)=\frac{\left(Ws-Wd\right)}{Wd}\cdot 100$$

where *Ws* is the swelled strip weight and *Wd* is the dried strip weight. The analyses were carried out at least in triplicate to estimate the inherent variability of each determination.

### 2.5. Mechanical Evaluation

#### 2.5.1. Folding Endurance

The bending strength of film formulations was evaluated by repeatedly folding the samples over the same place to break or reaching 300 folds. The number of times the sample could be folded without rupture indicated the value of bending strength [21,24]. At least three replicates of each film formulation were evaluated.

#### 2.5.2. Tensile Analysis

The mechanical characteristics of film formulations were evaluated using a TA-XTplus texture analyzer (Stable Micro Systems, Surrey, UK) in tension mode. The resistance to applied tension, Young’s modulus, force required for breaking, and elongation (maximum distance traveled until braking of the sample) of samples were determined [18,25]. Briefly, the samples were cut 50 mm in length and 10 mm in width, with 15 mm of each end of the sample in contact with the base (plate) of the apparatus, so that 20 mm was exposed. One of the tensile grips (ranging 35 × 35 mm) was fixed to the stationary base and the other to the travelling arm that moved at a speed of 2 mm/s up to the sample rupture. Young’s modulus, the forces of maximum tension (T_max_) and of fracture (F) at breakpoint were determined. The analyses were carried out at least in three replicate samples to estimate the inherent variability of each determination.

### 2.6. Ex Vivo Evaluation of Mucoadhesive Properties

The analysis of mucoadhesive properties of film formulations was accomplished using a TA-XTplus texture analyzer (Stable Micro Systems, Surrey, UK) in tension mode [26]. The mucoadhesive force of each sample was evaluated using the force required to remove the porcine buccal mucosa from the formulation. The mucosal tissue was obtained from pigs (white, young, and recent sacrificed) originated from a local slaughterhouse (authorized by the Brazilian Ministry of Agriculture for human consumption). Porcine buccal mucosa samples were cleaned with phosphate saline buffer (PSB) and prepared with an area of 132.73 mm^2^, using a surgical scalpel, whilst taking care to avoid the use of samples displaying wounds or bruises. The mucosal tissue was then horizontally attached to the lower termination of the probe (cylindrical, P/6), using double sided adhesive tape. A film sample was placed on a support at the bottom, and a downward force (0.03 N) was applied for 5 s, ensuring close contact between the mucosal tissue and the sample. The probe was moved upward at a constant velocity (10 mm/s), and the force required to detach the mucosa from the surface of the film sample was determined as the resultant force-time plot. The analyses were performed at least in three replicate samples of each film formulation [20,27].

### 2.7. Water Vapor Permeability

The film samples were cut to appropriate dimensions and mounted on a glass cylindric cell containing 10 mL of purified water. The charged cell was weighed and placed in pre-equilibrated desiccator (0% relative humidity) and maintained at temperature of 25 °C. After 24, 48, 72, 96 and 120 h, the cells were reweighed, and the water amount permeated through the film was determined from the weight loss from the assembled cell. The water vapor permeability (WVP, g/h·mm^2^) was calculated using the following equation [22,28,29]:

(3) $$\mathrm{WVP}=\frac{m}{t\cdot A}$$

where *m* is the permeated water weight (g), *t* is the time (h), and *A* the area of the film sample (mm^2^). All analyses were carried out at least in triplicate to estimate the inherent variability of each determination.

### 2.8. In Vitro Evaluation of Tramadol Release Profile

The analysis of tramadol release profile from film formulations was accomplished using a modified Franz’s cell-based apparatus, consisting of a cylindrical glass cell, and with a total capacity of 50 mL [20,30]. The temperature of analysis was 37.0 ± 0.5 °C controlled using a thermostatic bath. The dissolution medium was purified water (20 mL); the sink conditions were ensured, and constant magnetic stirring was applied. Moreover, the cellulose acetate membrane with 0.45 μm pores was used as a support (12,400 MWCO; Sigma-Aldrich, Sao Paulo, Brazil), which was allowed to stand in purified water for at least 24 h. At predetermined time intervals (15 min, 30 min, 1 h, 2 h, 4 h, and 6 h), a sample of 2.0 mL was withdrawn and the volume was replaced with purified water. The analysis was performed in at least six replicates of each film formulation. The tramadol content was determined by spectrophotometry, using a validated method [31]. Briefly, an analytical curve (calibration curve) was obtained using six replicates with dilutions of 5.0, 10.0, 35.0, 45.0, 65.0, 85.0, and 100.0 µg/mL, and was analyzed at a wavelength of λ = 271 nm [32].

### 2.9. Statistical Analysis

The physicochemical characteristics of film formulations were statistically compared using a three-way analysis of variance (ANOVA). The individual differences between means were identified using Tukey’s honestly significant difference test. Moreover, the effects of the different polymers’ amounts on swelling index, mechanical and mucoadhesive characteristics of formulations were statistically compared using DoE. Therefore, a polynomial model that correlates the independent variables and the response is further described by the following Equation (4):

y = b_0_ + b_1_X_1_ + b_2_X_2_ + b_3_X_3_ + b_12_X_1_X_2_ + b_13_X_1_X_3_ + b_23_X_2_X_3_ + b_123_X_1_X_2_X_3_ (4)

where y is the response, b_0_ is the arithmetic mean response, b_1_ − b_3_ are the estimated coefficients for X_1_ − X_3_, respectively, and b_12_ − b_23_ are the estimated coefficients for interaction terms.

Significant differences were considered when *p* < 0.05, and the Statistica 10.0 software (StatSoft Company, Tulsa, OK, USA) was utilized.

## 3. Results and Discussion

### 3.1. Film Preparation

Considering the factorial design and the solvent casting method, it was possible to obtain film for all combinations of polymeric concentration. Formulations were easily removed from the molds. Initially, the macroscopic characteristics of the films were evaluated, considering flexibility, integrity, homogeneity (thin and thick parts), opacity (due to the presence of EC), and the presence of small bubbles. All these features showed varied intensities among the formulations.

They showed integrity and flexibility; however, the formulations containing the highest amount of EC (17.5%) displayed a greater amount of bubbles, as well as greater opacity, in addition to greater stiffness. These results can be found in some formulations where EC makes up more than 15% in the formulation [33]. The films containing the highest PVP concentration were more malleable and displayed greater tactile adhesiveness. These characteristics may be due to PVP’s swelling property in an aqueous medium, with the capacity to retain more than 0.5 mol of water per mol of polymer, which increased with the application of body heat [34].

### 3.2. Thickness and Density

The results of thickness and density of the film samples are displayed in Table 2. It was observed that the density did not show a significant statistical difference (*p* > 0.05). Film formulations displayed density values from 0.00015 to 0.00021 g/mL. However, the thickness of film formulations was significantly different (*p* < 0.05), probably due to the different amounts of each polymer in each preparation. It was observed that the increase in the polymeric amounts increased the film thickness and mass, mainly for the formulations containing highest level of one or more polymers (e.g., F2, F4, F5, F6, F7 and F8).

### 3.3. Swelling Index (SI)

The *SI* evaluates the hydration capacity of polymeric matrixes [35] and Table 2 shows the moisture uptake capacity of film formulations. PVP has hydrophobic characteristics, which makes it difficult for water to enter the polymer chains and, consequently, for the chains to disperse in an aqueous medium. On the other hand, PVA and EC of the aqueous dispersion are hydrophilic, facilitating the interaction with water [20,36]. The *SI* analysis also allows evaluating the properties that exert an effect on the control of the drug release kinetics. Due to the films tested being composed of two polymers with the hydrophilic characteristics of fast dissolution in an aqueous medium and fast disintegration, compared to only one hydrophobic polymer, during the analysis it was not possible to obtain the *SI* data at all times initially foreseen, only for the time 60 s. Despite the formulations being able to display different affinities to water due to their dependence on their polymeric compositions, a significant effect of independent variables in *SI* of film formulations was not observed (*p* > 0.05).

### 3.4. Mechanical Evaluation

The mechanical characteristics of film formulations are displayed in Table 3. This evaluation is useful to assess the basic film-forming properties of new materials, as well as to predict their usefulness for pharmaceutical use (e.g., platform for drug delivery) [19,35]. It was possible to fold the film samples more than 300 times without breakage, showing that both the compositions resulted in good folding characteristics [19]. This result demonstrates that the differences in the concentration of polymers in the films did not influence their resistance (*p* > 0.05). This is a desirable feature for film formulations, since, when administered, they cannot be broken due to impacts from external factors that they may suffer, such as pressure for administration, tongue friction or mouth movement during speech [19,35,37].

The composition of each film formulation influenced its mechanical tensional properties (Table 3). Young’s modulus is an indicator of film stiffness, in which the higher the values, the greater the stiffness. It is defined as the ratio between the applied stress (force per unit area) and the resulting elongation (relative variation in sample length) [18]. In this study, Young’s modulus values were dependent on PVP and EC concentrations, as well as the interaction between the polymers PVA-EC and PVO-EC (*p* < 0.05). Equation (4) was used to calculate the values of the estimate coefficients for Young’s modulus, and the resulting Equation (5) is described below:

y = 2.1787 + 1.0160 X_2_ + 0.9020 X_3_ + 1.5170 X_1_X_3_ − 0.8218 X_2_X_3_ + 1.1048 X_1_X_2_X_3_ (5)

**Figure 1:**
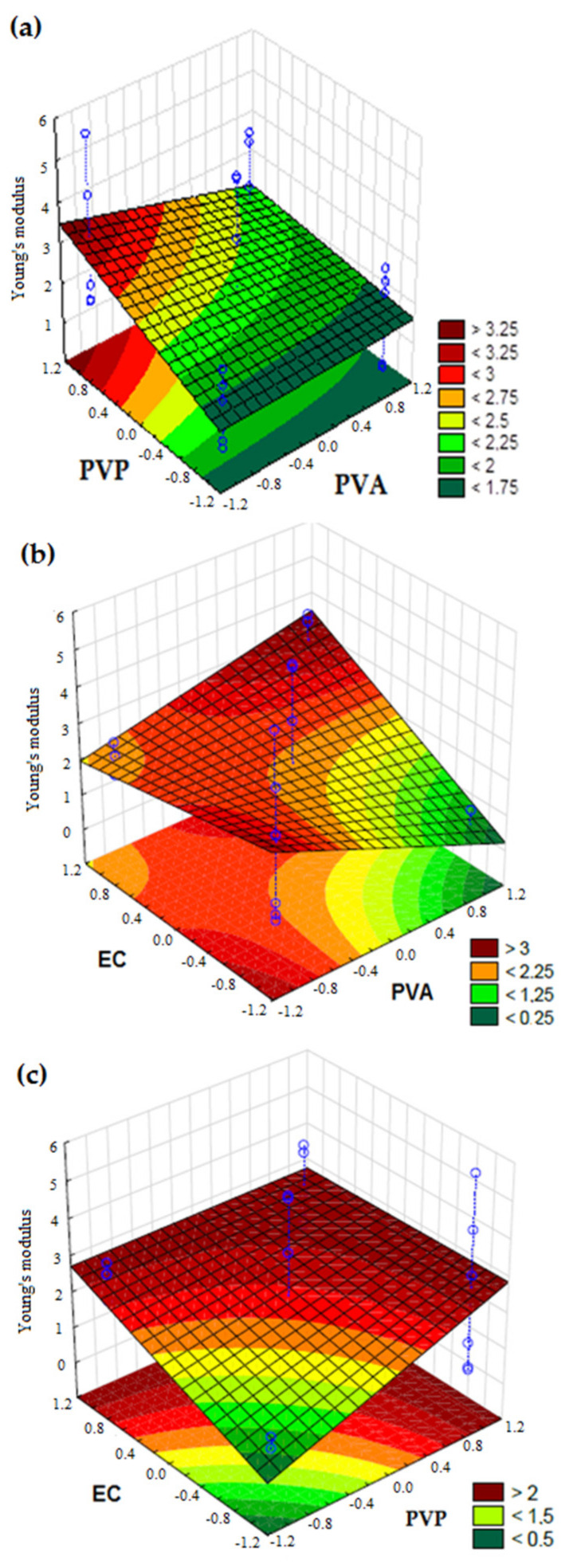
Response surface plots of mechanical tensional characteristic (Young’s modulus) of film formulations at 25 °C as a function of: (**a**) PVA (X_1_) and PVP (X_2_); (**b**) PVA (X_1_) and EC (X_3_); (**c**) PVP (X_2_) and EC (X_3_). The color scale is indicated in each figure and shows the isoparametric values.

The PVA did not exert a significant influence (*p* > 0.05), because this polymer does not provide stiffness to the film. However, as the PVP or EC amounts increased, the Young modulus of films increased (*p* < 0.05) (Figure 1). The increased amounts of EC significantly increase the Young modulus of formulations, except when analyzing the effect of EC and PVP, where the interaction was negative. However, when the interaction among the three polymers was analyzed, the result was positive.

The maximum tensile stress is indicated by the maximum point of a strain–stress curve. That is, it is the maximum tension a material can withstand when stretched before breaking. The mechanical behavior of the films can vary according to the structural characteristics of the polymers, such as molar mass, presence of polar groups, cross-links, among others. The tension is dependent on the presence of surface defects, in addition to the temperature of the test and the material [38]. Generally, the greater the number of polymers in the system, the greater the film’s mechanical strength [39]. The presence of a larger number of polymers conduces a smaller distance among the molecules that can lead to a greater interaction among the film layers and, consequently, a high resistance. In contrast, film formulations containing a smaller number of polymeric molecules can display a lesser interaction among the molecules and, consequently, a lower resistance to rupture [18,39].

In this sense, the formulations containing the lowest (F1) and highest (F8) levels of both three polymers would display the smallest and greatest values of the mechanical tensional properties, respectively. However, the highest value for F_max_ was observed for formulation F4 (level +1 for PVA), while the lowest one was observed for F7 (level +1 for PVA and PVP). Moreover, formulation F2 (level +1 for EC) displayed the highest value for fracture (F = 34.59 ± 2.07 N), while F7 displayed the lowest one (F = 5.94 ± 1.09 N).

Equation (4) was used to calculate the values of the estimate coefficients for F_max_, and the resulting Equation (6) is described below:

y = 18.4359 + 7.7514 X_1_ + 10.4164 X_3_ + 8.5479 X_1_X_3_ (6)

**Figure 2:**
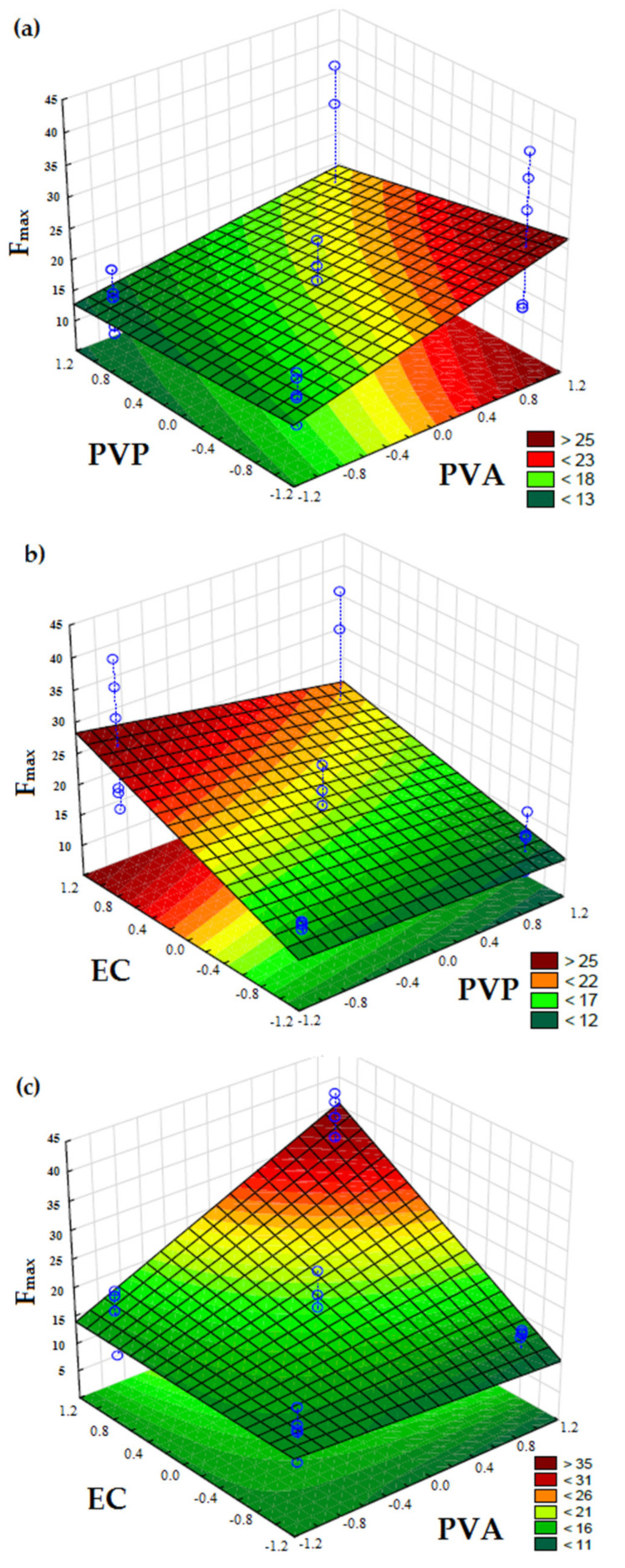
Response surface plots of mechanical tensional characteristic (F_max_) of film formulations at 25 °C as a function of: (**a**) PVA (X_1_) and PVP (X_2_); (**b**) PVP (X_2_) and EC (X_3_); (**c**) PVA (X_1_) and EC (X_3_). The color scale is indicated in each figure and shows the isoparametric values.

The F_max_ significantly increased as the PVA and EC amounts increased in the film formulations (*p* < 0.05); however, the influence of PVP was not significant (*p* > 0.05). These effects can also be observed in Figure 2.

The formulations F4 and F8 displayed the highest F_max_ values, 35.51 and 28.80 N, respectively. Both film formulations were composed of the highest amounts of PVA and EC polymers. In contrast, the lowest values of F_max_ were found for formulations F7 (10.95 N), F6 (13.52 N) and F5 (14.20 N), which were composed of the lowest level of EC. Thus, PVA and EC are the polymers that confer resistance to the film, and it can also be inferred that there is an interaction between them. PVA and PVP have shown to result films with good mechanical properties [18,19,34]. These two polymers can interact due to the hydrogen bonds formed between the hydroxyl group of PVA and the carboxyl group of PVP [40]. The addition of small amounts of PVP to PVA results in an improvement in the stability of this polymer blend through the inter-chain bonding and also increases crystal clarity and decreases PVA degradation [41,42]. In this study, formulations F5 to F8 are composed of the highest amount of PVP, and it is also necessary to consider the presence of another polymer (EC). Thus, EC can compete with the PVP to make the interactions (the hydrogen bonds) with the PVA. The same behavior was observed for Young’s modulus.

The fracture or break due to the tension applied is the maximum stress that generates the sample rupture. The values for fracture were dependent on the EC concentration (*p* < 0.05) and on the interaction between EC and PVA (*p* < 0.05), as described by the following Equation (7):

y = 12.5761 − 5.8176 X_2_ + 6.6400 X_3_ + 5.7130 X_1_X_3_ (7)

**Figure 3:**
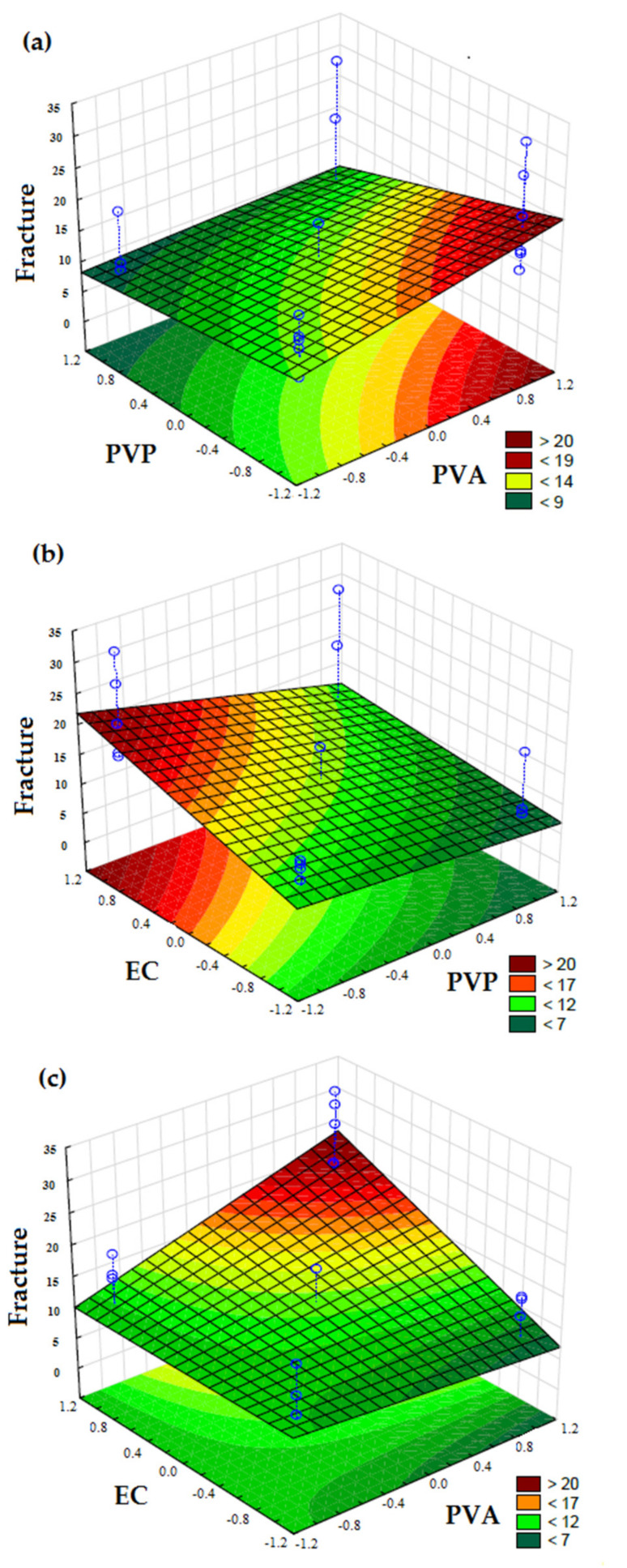
Response surface plots of mechanical tensional characteristic (Fracture) of film formulations at 25 °C as a function of: (**a**) PVA (X_1_) and PVP (X_2_); (**b**) PVP (X_2_) and EC (X_3_); (**c**) PVA (X_1_) and EC (X_3_). The color scale is indicated in each figure and shows the isoparametric values.

EC was the polymer that most influence on film strength and hardness. The concentration of PVP showed a significant effect, but this was negative. Thus, as the PVP amount increased the resistance to fracture significantly decreased (*p* < 0.05) (Figure 3). As observed for the previous mechanical characteristics, the polymers PVA and EC resulted in films with more resistance.

### 3.5. Mucoadhesive Properties

A pharmaceutical dosage form designed for the oral mucosa administration must be retained at the site for a suitable period of time for drug release. This time may be enough for a fast or prolonged delivery, but for the optimized drug availability. For this, the film must have an adequate mucoadhesive characteristic [11,43]. Thus, the properties of the polymer, such as the presence of specific functional groups and the flexibility of its chains, are essential for the establishment of the mucoadhesive process [14]. The results obtained for mucoadhesive force of film formulations are displayed in Table 3.

The mucoadhesive strength of the films was mainly dependent on the concentration of PVA and EC in the films (*p* < 0.05); however, the PVA effect was negative, indicating that the increase in this polymer decreased the mucoadhesive force of formulations, as described by the following Equation (8):

y = 0.0902 − 0.0304 X_1_ + 0.0168 X_3_ − 0.0393 X_1_X_3_ (8)

**Figure 4:**
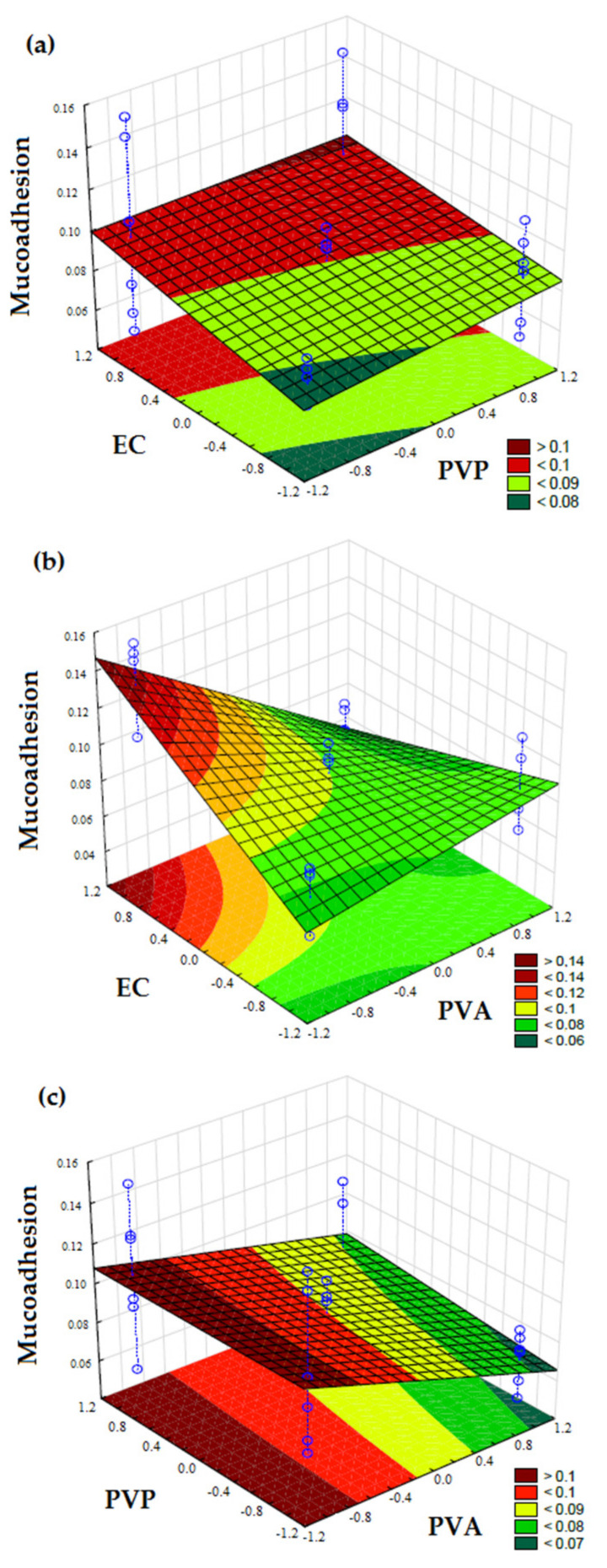
Response surface plots of mucoadhesive force of film formulations as a function of: (**a**) PVP (X_2_) and EC (X_3_); (**b**) PVA (X_1_) and EC (X_3_); (**c**) PVA (X_1_) and PVP (X_2_). The color scale is indicated in each figure and shows the isoparametric values.

The increase in EC significantly increased the mucoadhesion of film formulations (*p* < 0.05) and PVP did not show significant effect (*p* > 0.05). Figure 4 shows the response surface plots of mucoadhesive force, indicating the mains effects of each polymer.

The film formulations presented similar mucoadhesive force from 0.061 to 0.135 N. The greater mucoadhesiveness were displayed by F2 (0.135 N), F6 (0.133 N) and F10 (0.100 N). The common variables between the F2 and F6 films are the low concentration of PVA and the highest EC concentration. Regarding the concentration of PVP, the formulation F2 had the smallest amount, while F6 had the largest one. The common concentration of PVA and EC in both films possibly allowed the PVP polymer to maintain its adhesiveness in the formulation, regardless of its concentration [14].

Film formulations F1, F4 and F8 displayed the lowest mucoadhesive strengths: 0.076, 0.061, and 0.068 N, respectively. The common variables between the F4 and F8 films are also the same PVA and EC amounts, both at the highest level. Due to the presence of hydroxyl groups of PVA and carboxylic groups of PVP, all formulations displayed mucoadhesive characteristics [11,14,18,20,43].

### 3.6. Water Vapor Permeability (WVP)

Considering the results obtained by design, the selected formulations were those containing the highest amounts of PVA, PVP and EC. However, despite the results of films with a greater amount of EC being promising, during their preparation there was difficulty in dispersing and homogenizing, generating films with a greater amount of bubbles and without uniformity. Thus, films with a higher amount of EC were not selected, and the formulations F3, F5, F7, and F9/F10 were considered for the next analysis.

The analysis of WVP allows evaluating the aqueous permeability rate of film, considering the percentage of water that passes per unit of film area and of known thickness, induced by a pressure gradient among two specific surfaces, of known relative humidity and temperature [44]. The use of insoluble materials, or materials with low solubility in water, leads to a film with low permeability within a wide range of humidity [21]. When the materials are soluble in water, the permeability rate increases, and composite films can have the advantage of bringing together the positive points of each of the materials used [20]. Permeability can be defined as a process in which vapor dissolves on one side of the film and diffuses to the other side. Factors such as morphology, density, chemical structure, crystallinity and polymeric orientation of the film can influence the water permeability rate as well as the type of solvent, plasticizer and drying rate [45].

Therefore, the WVP evaluation of film formulations enabled the determination of permeability rate in function of the different polymeric amounts of PVA, PVP and EC of selected films (Table 4).

The different concentrations of the polymers did not result in significant variations in WVP (*p* > 0.05). Although the results obtained are close, the formulation F7 displayed the highest values every day, indicating that it allowed the greatest passage of water among the relaxation points of the polymeric chains. This is the film that has the highest concentrations of the PVA and PVC polymers and the lowest concentration of EC. On the other hand, the formulations F3 and F5 displayed the lowest WVP values. These, in turn, have the lowest concentrations of PVP and PVA, respectively, which suggests that decreasing the amount of these polymers decreases the relaxation of polymer chains and consequently the transmission of water through the films [19]. As these selected films have the lowest amount of EC, it is possible to improve the PVA and PVP molecules interacting [40,41].

### 3.7. In Vitro Evaluation of Tramadol Release Profile

**Figure 5:**
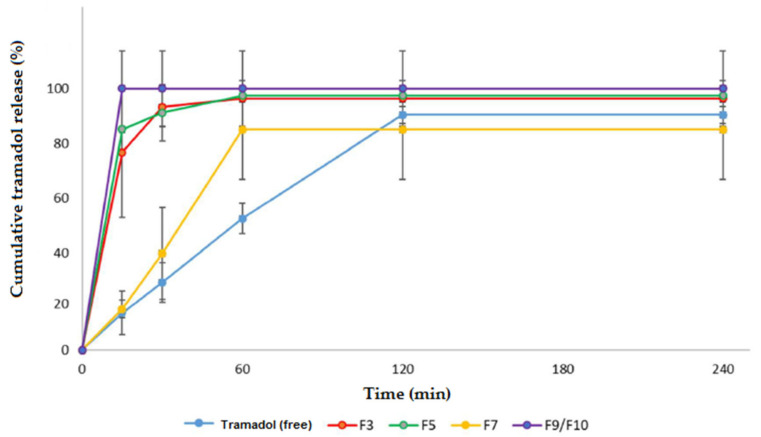
The effects of different amounts of polyvinyl alcohol (PVA), polyvinylpyrrolidone (PVP), and ethylcellulose (EC) on tramadol release from selected film formulations: F3, F5, F7, and F9/F10 and free tramadol. Each curve is the mean ± standard deviation of at least three analyses.

The in vitro evaluation of tramadol release profile is a fundamental step during the development of proposed mucoadhesive fast-dissolving film system. In this context, the analysis is performed with success when the experimental conditions (sink conditions, stirring and temperature) are appropriate, simulating the in vivo conditions [21,36,46]. Tramadol hydrochloride is readily soluble in water, which was used as dissolution media to investigate the influence of the technology applied on drug release. Thus, the film formulations were analyzed with controlled temperature and agitation, and the sink conditions were maintained. The results are displayed in Figure 5.

The formulation F5 and the central point (F9/F10) displayed the fastest tramadol release, and 100% of drug was released up to 15 min. Formulations F3 and F7 displayed slower release, and the total tramadol release was accomplished by 30 and 60 min, respectively.

The tramadol solution (free drug) displayed the slowest release, and it was totally released in approximately 120 min. The results obtained from the films, in comparison with the tramadol solution, showed a decrease in the drug release time; thus, we are able to infer that the polymeric platform used had an influence on the results in order to allow a faster drug release. It is noteworthy that these results were obtained in vitro and may indicate that the presence of polymers and applied technology helped in the faster dissolution of tramadol and its mass transfer to the dissolution medium through the cellulose acetate membrane.

These results are in agreement with the previous one, and indicate that the PVA, due to its characteristic of matrix formation, helps in the structuring and maintenance of the film, which allowed an increase in the drug release time. When the concentration of PVP also increased (for example, in the F7 film), this structuring became firmer, possibly due to the polymer blend formed by the hydroxyl groups of the PVA and PVP carbonyl groups [34]. It is important to emphasize that this F7 film had the lowest EC content. This composition made it possible to prolong the drug release time.

On the other hand, the concentration of EC was not taken into account, as it was at its lowest concentration in all films, except for the zero point. The F9/F10 formulations have a higher proportion of EC compared to other polymers, making the interaction between PVA and PVP difficult, as observed in the previous characterizations. Thus, at the central point, a matrix was formed that was more susceptible to the entry of the permeant (water) for the dissolution and consequent faster tramadol release. Thus, the fast tramadol release was possibly due to EC interference. The EC dispersion used (Surelease^®^) is an aqueous compound containing ethylcellulose, ammonium hydroxide, medium chain triglycerides and oleic acid [47], and also containing about 25% (m/m) of plasticizers, which may have contributed to the rapid release of TrHC from the formulations.

Studies have shown the solubility enhancement of poorly soluble APIs, using platforms composed of hydrophilic polymers, and electrospinning represents a useful technological strategy [48]. However, tramadol hydrochloride has a low molecular weight (299.83 g/mol) and log partition coefficient (logP) in n-octanol-water of 1.35 (at pH 7), which are advantageous characteristics from the point of mucosal absorption. Following oral administration, tramadol is rapidly and almost completely absorbed, with a bioavailability of 75% due to the first-pass metabolism (20–30%), and shows peak plasma concentrations at two hours [49,50]. Thus, the fast drug release enabled by F9/F10 formulations represents a useful means to improve the buccal absorption rate of tramadol. In addition, the effect of saliva’s flush in the buccal cavity, the swallowing of the dissolved tramadol and also the first-pass effect probably will be low.

## 4. Conclusions

The use of a 2³ factorial design, with repetition at the central point, was extremely important to help define the best polymer concentrations, to ensure a good performance during the formulation preparation steps and to identify the influence of each component in the formulation. The films were homogeneous and had high mechanical strengths and mucoadhesion. These attributes are desirable, as they contribute to the permanence of the formulation at the administration site and to greater tramadol availability. The most promising film formulations were those prepared with the greatest amount of PVA, PVP and EC. However, EC showed difficulty in dispersion and homogenization, generating films with a greater amount of bubbles and without uniformity. Thus, films with a higher amount of EC were disregarded, highlighting that films should not contain more than 15% EC. The applied technology promoted the obtainment of pharmaceutical films with adequate mechanical characteristics and mucoadhesive properties, in addition to providing fast drug release. Therefore, it was possible to develop mucoadhesive fast-dissolving films with suitable mechanical characteristics to be used as platform systems for oral administration for a faster drug action. They constitute platforms and strategies to improve the therapy of tramadol with regard to availability at the site of application, considering the necessity of rapid pain relief, and show potential for in vivo evaluation.

## Figures and Tables

**Table 1:** Matrix of factorial design matrix 2^3^ (plus two center points) for formulations containing polyvinyl alcohol (PVA), polyvinylpyrrolidone (PVP), ethylcellulose (EC) and tramadol (10%, *w*/*w*) *.

Independent Variables/Factors (%, *w*/*w*) **	Levels		
	Low	Central	High
	(−1)	(0)	(+1)
X_1_ = PVA	50	60	70
X_2_ = PVP	10	20	30
X_3_ = EC	12.5	15	17.5
**Standard run (formulations)**	**X_1_**	**X_2_**	**X_3_**
F1	-	-	-
F2	-	-	+
F3	+	-	-
F4	+	-	+
F5	-	+	-
F6	-	+	+
F7	+	+	-
F8	+	+	+
F9 (C)	0	0	0
F10 (C)	0	0	0

* The dryness film; ** the liquid dispersion; C = center point.

**Table 2:** Physicochemical characteristics (thickness, mass, density, and swelling index) of film formulations composed of polyvinyl alcohol (PVA), polyvinylpyrrolidone (PVP), ethylcellulose (EC) and tramadol (10%, *w*/*w*). The results represent the mean (± standard deviation) of at least three of the replicate samples analyzed.

Formulations	Thickness (mm)	Mass (g)	Density (g/mL) *	*SI* (%) *^,^**
F1	0.11500 ± 0.05263 ^a^	0.01435 ± 0.00690 ^a^	0.00014 ± 0.00007	156.88 ± 73.63
F2	0.18275 ± 0.08176 ^b^	0.01610 ± 0.00748 ^a^	0.00016 ± 0.00007	92.52 ± 17.05
F3	0.11750 ± 0.05857 ^a^	0.01550 ± 0.00706 ^a^	0.00016 ± 0.00007	130.82 ± 64.96
F4	0.15250 ± 0.06870 ^c^	0.01658 ± 0.00748 ^b^	0.00017 ± 0.00007	147.50 ± 53.98
F5	0.13500 ± 0.06301 ^c^	0.01658 ± 0.00743 ^b^	0.00017 ± 0.00007	148.75 ± 39.16
F6	0.15000 ± 0.07141 ^c^	0.01818 ± 0.00831 ^c^	0.00018 ± 0.00008	100.68 ± 49.04
F7	0.16750 ± 0.07701 ^b^	0.02005 ± 0.00898 ^c^	0.00020 ± 0.00009	147.50 ± 10.85
F8	0.17250 ± 0.07950 ^b^	0.02148 ± 0.00962 ^c^	0.00021 ± 0.00010	131.99 ± 63.82
F9 (C)	0.10750 ± 0.04827 ^a^	0.01440 ± 0.00648 ^a^	0.00014 ± 0.00006	89.91 ± 10.28
F10 (C)	0.11500 ± 0.05167 ^a^	0.01478 ± 0.00663 ^a^	0.00015 ± 0.00007	80.11 ± 11.24

* No significant statistical difference (*p* > 0.05); ** after 60 s; for each physicochemical characteristic, the means with different letters (^a^, ^b^ and ^c^) indicate significant statistical differences (*p* < 0.05).

**Table 3:** Mechanical and mucoadhesive properties of film formulations composed of polyvinyl alcohol (PVA), polyvinylpyrrolidone (PVP), ethylcellulose (EC) and tramadol (10%, *w*/*w*). The results represent the mean (± standard deviation) of at least three of the replicate samples analyzed.

Formulation	Young’s Modulus (MPa)	F_max_ (N)	F (N)	Folding Endurance (Times) *	Mucoadhesive Force (N)
F1	1.39 ± 0.25	20.19 ± 2.27	16.97 ± 2.28	>300	0.0760 ± 0.0120
F2	2.13 ± 0.38	18.20 ± 1.87	34.59 ± 2.07	>300	0.1350 ± 0.0270
F3	0.39 ± 0.02	15.43 ± 0.32	13.57 ± 1.72	>300	0.080 ± 0.0050
F4	2.52 ± 0.31	35.51 ± 4.60	26.32 ± 5.83	>300	0.0610 ± 0.0120
F5	4.28 ± 1.37	14.20 ± 4.76	11.79 ± 5.72	>300	0.0790 ± 0.0200
F6	1.73 ± 0.22	13.52 ± 5.35	8.31 ± 1.42	>300	0.1330 ± 0.0150
F7	0.61 ± 0.41	10.95 ± 3.11	5.94 ± 1.09	>300	0.0930 ± 0.0260
F8	3.31 ± 0.73	28.80 ± 11.66	17.90 ± 13.55	>300	0.0680 ± 0.0090
F9 (C)	4.33 ± 0.86	15.22 ± 8.60	7.03 ± 1.87	>300	0.0750 ± 0.0070
F10 (C)	1.69 ± 0.28	20.97 ± 1.59	7.40 ± 5.61	>300	0.1000 ± 0.0050

F_max_ = Maximum tension; F = Fracture; * no significant statistical difference (*p* > 0.05).

**Table 4:** Water vapor permeability (WVP) as a function of time (h) of selected film formulations composed of polyvinyl alcohol (PVA), polyvinylpyrrolidone (PVP), ethylcellulose (EC) and tramadol (10%, w/w).

Formulations	WVP (× 10^−5^ g/h.mm^2^)				
	24 h *	48 h *	72 h *	96 h *	120 h *
F3	1.5624 ± 0.0389	1.4982 ± 0.0387	1.4390 ± 0.0398	1.3919 ± 0.0349	1.4490 ± 0.0432
F5	1.5481 ± 0.0435	1.5169 ± 0.0496	1.4920 ± 0.0398	1.4209 ± 0.0263	1.4710 ± 0.0489
F7	1.7823 ± 0.0590	1.6190 ± 0.0280	1.52610 ± 0.0476	1.4972 ± 0.0354	1.6200 ± 0.0374
F9 (C)	1.6723 ± 0.0489	1.5163 ± 0.0387	1.4910 ± 0.0452	1.4961± 0.0411	1.5900 ± 0.0399
F10 (C)	1.6340 ± 0.0378	1.5129± 0.0412	1.4730 ± 0.0431	1.501± 0.0398	1.5410 ± 0.0452

* No significant statistical difference (*p* > 0.05).
